# Effects of a Single Session of High Intensity Interval Treadmill Training on Corticomotor Excitability following Stroke: Implications for Therapy

**DOI:** 10.1155/2016/1686414

**Published:** 2016-09-25

**Authors:** Sangeetha Madhavan, James W. Stinear, Neeta Kanekar

**Affiliations:** ^1^Department of Physical Therapy, University of Illinois at Chicago, Chicago, IL, USA; ^2^Department of Exercise Sciences, University of Auckland, Auckland, New Zealand

## Abstract

*Objective*. High intensity interval treadmill training (HIITT) has been gaining popularity for gait rehabilitation after stroke. In this study, we examined the changes in excitability of the lower limb motor cortical representation (M1) in chronic stroke survivors following a single session of HIITT. We also determined whether exercise-induced changes in excitability could be modulated by transcranial direct current stimulation (tDCS) enhanced with a paretic ankle skill acquisition task.* Methods*. Eleven individuals with chronic stroke participated in two 40-minute treadmill-training sessions: HIITT alone and HITT preceded by anodal tDCS enhanced with a skill acquisition task (e-tDCS+HIITT). Transcranial magnetic stimulation (TMS) was used to assess corticomotor excitability of paretic and nonparetic tibialis anterior (TA) muscles.* Results*. HIIT alone reduced paretic TA M1 excitability in 7 of 11 participants by ≥ 10%. e-tDCS+HIITT increased paretic TA M1 excitability and decreased nonparetic TA M1 excitability.* Conclusions*. HIITT suppresses corticomotor excitability in some people with chronic stroke. When HIITT is preceded by tDCS in combination with a skill acquisition task, the asymmetry of between-hemisphere corticomotor excitability is reduced.* Significance*. This study provides preliminary data indicating that the cardiovascular benefits of HIITT may be achieved without suppressing motor excitability in some stroke survivors.

## 1. Introduction

High intensity interval treadmill training (HIITT) is gaining popularity in the fitness industry and as a promising stroke rehabilitation protocol to improve cardiovascular and motor outcomes [1]. It involves short bursts of high effort alternated with longer recovery periods to maximize efficacy of training [2]. Treadmill training at high speeds results in improved overground gait speeds, facilitates a more normal walking pattern, and improves cardiovascular efficiency [2–5]. Although previous studies have examined the effects of HIITT on gait parameters and cardiovascular outcomes after stroke, no study has examined the effects of HIITT on corticomotor excitability after stroke. Despite the promising effects of HIITT (noted above), it is not known whether HIITT induces central fatigue in the corticomotor system. This is an important question to answer because people with stroke have a high reported incidence of central fatigue [6–8]. Insufficient central drive and an imbalance of between-hemisphere symmetry of corticomotor exitability (CME) are a well-established benchmark after stroke [9, 10]. People with stroke exercising at a high intensity may develop central neural fatigue that could compromise their ability to drive descending motor output [11–13]. Hence, the primary purpose of this study was to examine the short-term changes in CME of the lower limb M1 following a single session of HIITT. We hypothesized that a single session of HIITT would decrease CME of paretic lower limb muscles and create a further imbalance of between-hemisphere cortical excitability in stroke survivors. We also wanted to know if increasing lower limb corticomotor excitability prior to a session of HIITT could mitigate these changes. We chose transcranial direct current stimulation (tDCS) combined with a visuomotor ankle-tracking task that we had previously demonstrated to be a robust facilitator of lower limb skill acquisition and CME assessed in the paretic limb tibialis anterior (TA) muscle [14]. At the risk of limiting the conclusions that could be drawn from the study, we did not include additional sessions of skill acquisition alone and tDCS alone for this group of people with stroke and included two sessions: HIITT alone and HIITT plus anodal tDCS enhanced with ankle-tracking (e-tDCS+HIITT). The goal of the present study was, therefore, to provide preliminary data to inform future research that would examine in more detail whether the physiological and functional benefits of HIITT are limited by the induction of central fatigue.

## 2. Methods

### 2.1. Participants

Eleven individuals with chronic stroke participated in the study (see Table 1 for demographics). This was a carefully selected homogenous sample as these individuals had participated in a previous nonintervention study in the laboratory. These individuals were selected because transcranial magentic stimulaion (TMS) induced motor evoked potentials (MEPs) could be induced in their paretic leg muscles and demonstrated 5 degrees or more of active paretic ankle dorsiflexion necessary to perform our tDCS-enhanced ankle motor task. In addition, participants were able to walk independently (with or without an assistive device) for at least 10 minutes, a criterion necessary to fully participate in HIITT. Participants did not have contraindications to TMS, such as metallic implants in the head region, a history of seizures, implanted cardiac pacemakers, and medications known to alter central nervous system excitability. The Mini Mental State Examination (MMSE) was used to assess cognitive impairment, and those with a score of less than 24 (out of 30) were excluded. All the participants signed a written informed consent form approved by the Institutional Review Board of the University of Illinois at Chicago.

### 2.2. Experimental Protocol

Each individual participated in two treadmill training sessions, HIITT alone and e-tDCS+HIITT, the sessions being one week apart and pseudorandomized to avoid order effects. Prior to and at the end of each session, participants performed two trials of the overground 10-meter fast walking test (using their walking aid, e.g., cane and ankle foot orthosis if required). Baseline (pre-) and post-training corticomotor excitability measures were obtained for TA muscles bilaterally using single-pulse TMS. During the e-tDCS+HIITT session, participants received anodal tDCS over the lesioned lower limb M1 prior to treadmill training. During anodal tDCS, participants performed a visuomotor tracking task with their paretic ankle for 15 minutes while receiving tDCS. In order to match the e-tDCS+HIITT session duration with the HIITT only session, participants in the latter were asked to remain seated quietly for fifteen minutes prior to treadmill training. Blood pressure (BP) and heart rate (HR) were monitored during the fifteen minutes of tDCS and during the fifteen minutes of rest. No differences in BP and HR were noted during these seated sessions.

### 2.3. Electromyography (EMG)

Muscle activity was recorded bilaterally from the TA using surface Ag/AgCl electrodes (Delsys Bagnoli 8, MA, USA) placed over the muscle belly after standard skin preparation. The ground electrode was placed over C7 spinous process. Two maximum voluntary isometric contractions (MVIC) were obtained for each muscle with the participants seated on a chair with knee flexed to 90° and the ankle in the neutral position and stabilized by a metal bar placed firmly and comfortably over the foot and secured to a wooden board to prevent movement of the foot. Pre- and post-training TMS measures were obtained, while participants produced a target EMG contraction corresponding to 10% MVIC for each muscle. EMG data were sampled at 2000 Hz, with a gain of 1000, and band-pass filtered (10–500 Hz). Spike2 software (Cambridge Electronic Design, Cambridge, UK) was used to collect the EMG data.

### 2.4. Transcranial Magnetic Stimulation

Single-pulse TMS at 0.25 Hz was delivered using a Magstim 200 stimulator (Magstim, Dyfed, Wales, UK) via a double-cone coil (diameter 110 mm) oriented to induce a posterior-anterior current flow in the cortex. Spike2 software was used to trigger the stimulator and also to record the trigger pulses. TMS was used to generate MEPs with the coil positioned contralateral to the TA muscle being tested (the nonparetic TA was tested first). A tightly fitted linen cap was placed on the participant's head and the position of the vertex (intersection of the lines connecting the nasion-inion and the two tragi) was marked. The TMS coil was placed on the cap at the vertex and then moved systematically to determine the hotspot for each muscle. The location of the hotspot for each TA was marked on the cap and the position was checked constantly by the experimenter during data collection to ensure that the coil was in the same position throughout. During TMS, participants were given visual feedback of muscle activity and asked to maintain a tonic contraction of the TA that represented 10% of MVIC. Active motor threshold (AMT) was determined as the stimulus intensity resulting in identifiable MEPs of at least 0.4 mV peak-to-peak in 50% of ten successive trials from the contralateral TA [14, 15]. Responses were obtained at seven TMS intensities corresponding to 80–140% of AMT (randomized order) to generate a recruitment curve for each TA muscle. Six MEPs were recorded for each intensity at pre- and post-training. The same intensities were used to collect post-training responses (five minutes after treadmill training). For the e-tDCS+HIITT session, the hotspot of the paretic TA was first determined by placing the TMS coil directly over the scalp. The active tDCS electrode was then placed on the scalp, over which the cap was tightly fixed, and the hotspot procedure repeated.

### 2.5. Anodal tDCS

tDCS was delivered using a constant current stimulator (Chattanooga Iontophoresis System, Hixon, TN, USA) via an 8 cm^2^ oblong saline-soaked sponge anode placed directly on the scalp over the hotspot for the paretic leg M1 and a self-adhesive carbonized reference cathode (35 cm^2^) placed on the forehead above the contralateral orbit. A 1 mA current was applied for fifteen minutes, while the participant performed the motor training task [16].

For the motor training task, participants performed visuomotor tracking with their paretic ankle. Details of the motor training task have been previously reported [14, 15, 17, 18]. In brief, participants were required to track, as accurately as possible, a computer-generated sinusoidal waveform using a voltage generated by an electrogoniometer attached to their paretic ankle, while they performed continuous ankle dorsiflexion and plantarflexion movements for 15 minutes. They were given 1 minute of rest after every 4 minutes of tracking. Our previous study found that tDCS strongly facilitated corticomotor excitability and enhanced fine motor control of the ankle approximately 3-fold compared with tracking practice alone [14]. This finding is supported by other research that demonstrated that tDCS during a motor task is more effective in increasing corticomotor excitability than tDCS administered during rest [18, 19].

### 2.6. Treadmill Training

**Figure 1:**
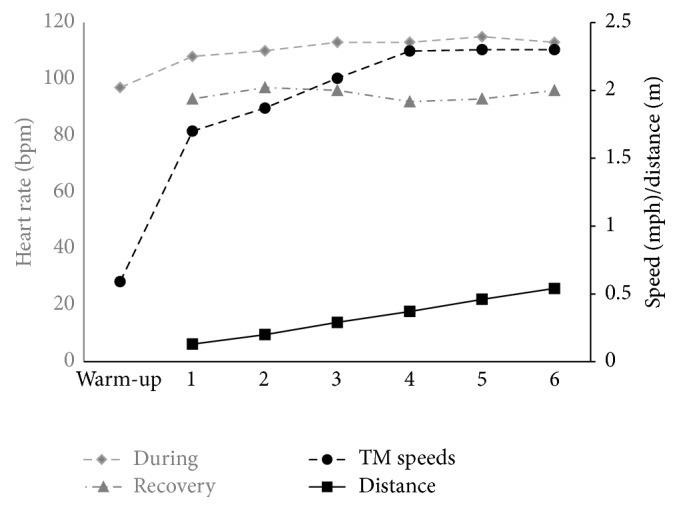
Representative example from one participant (number 2) during HIITT showing heart rate during training (gray diamonds), heart rate during recovery (gray triangles), treadmill speeds achieved at each interval (black circles), and composite distance (black squares). Heart rate (beats per minute) is represented on the primary left *y*-axis. Speed (miles per hour) and distance (miles) are presented on the secondary right *y*-axis. *x*-axis represents the time scale during treadmill training: 5 minute warm-up, the six walking intervals that the participant achieved during the session or in the case of the recovery HR, and the six recovery intervals that the participant needed to restore the HR to baseline.

All participants participated in the HIITT protocol. The training was modified based on the protocol used by Pohl and colleagues [3]. The protocol consisted of 40 minutes (5 minutes warm-up, 30 minutes walking, and 5 minutes cooldown) of treadmill walking with a structured increase in walking speed. The treadmill was set at 0% incline and participants were fitted in a harness for safety with no body weight support. Participants were given no assistance with walking. Heart rate (HR) and rate of perceived exertion (RPE) using the modified Borg Scale were continuously monitored. Age-predicted HR was determined using the formula (220 − age) and 80% of the age-predicted HR was set as the upper cut-off safety limit while increasing the belt speed. The maximum overground walking speed was determined from the 10 m fast walking test performed at the start of each session. The participants then conducted a five-minute warm-up on the treadmill at a speed half of their maximum overground walking speed. After the warm-up, the first speed-dependent training interval began. During a period of 2 minutes, the belt speed was increased, within the participant's tolerance, to the highest speed at which the participant could walk safely and without stumbling. At the end of the two-minute interval, this maximum achieved belt speed was held for ten seconds. This was followed by a recovery period when the participant walked at the warm-up speed until a time at which the participant's HR and RPE returned to the levels reached during the warm-up phase. If the participant maintained the speed and felt safe during the ten seconds at the end of the first-training interval, the speed was then increased by 10% during the next interval. This speed was again held for ten seconds at the end of the second interval and followed by another recovery period. During any fast walking phase, if the participant was unable to maintain the speed and felt unsafe, or the HR reached the cut-off safety limit, the speed was reduced by 10% for the next interval. At the end of thirty minutes of structured walking, a five-minute cooldown phase was provided. The participants performed the training similarly during HIITT and e-tDCS+HIITT sessions and were able to increase their speed by a factor of 3 to 5 during each session. After the forty minutes of treadmill training, participants were given five minutes to rest before TMS measures were taken. BP, HR, and RPE were recorded during this time. An example of one participant's treadmill training speeds, distance walked, and heart rate is provided in Figure 1.

### 2.7. Data Analyses

#### 2.7.1. Gait Speed

An average pre- and post-training gait speed was calculated from the two pre- and two post-10 m fast trials for each participant for each session. A change in gait speed was calculated for each individual.

#### 2.7.2. MEP Analyses

Spike2 software was used to analyze all MEP data. MEP amplitude was chosen as the primary measure to capture changes in corticomotor excitability. A MEP window was established for each muscle for each participant, for the pre- and post-training TMS trial during each session, by finding the onset and offset latencies of a large MEP in response to the highest TMS intensity (140% AMT). A window of identical width was set prior to the TMS stimulus to measure the tonic background contraction. The same MEP and background windows were then applied to analyze all the MEPs within a given session. MEP amplitude was calculated as the peak-to-peak magnitude of EMG activity within the MEP window and averaged across the six MEPs for each TMS intensity, each trial (pre and post), each muscle, and participant. The average MEP response was plotted against the corresponding stimulus intensity, and a linear function was used to fit this recruitment curve. The slope of this recruitment curve was calculated and a change in CME (gain) was determined for the nonparetic (NP) and paretic (P) TA muscles using the following equation:

(1) Percent change in CME=Postslope−PreslopePreslope∗100.

A physiological measure of interhemispheric symmetry of corticomotor excitability was calculated as follows: paretic slope/nonparetic slope. This ratio yields a value between 0 and 1 where values close to 1 indicate well-balanced interhemispheric symmetry and as values decrease towards zero, they indicate increasing levels of asymmetry [20]. This was used to establish the baseline level of interhemispheric symmetry for all participants.

### 2.8. Statistical Analyses

SPSS software (IBM software version 22, Armonk, NY) was used to perform all statistical analyses. A two-way repeated-measures ANOVA (session by time) was used to compare RPE, HR, and overground gait speeds between the two sessions. Four levels of time (warm-up, during training, cooldown, and after training) were analyzed for RPE and HR. Two levels of time (pre and post) were analyzed for overground gait speeds. Paired two-tailed *t*-tests were performed to compare differences between the baseline MEP slopes of the two sessions for each limb. Intraclass correlations (ICC) were also performed to examine test-retest reliability of baseline MEP slopes of the two sessions for each limb.

A two-way repeated-measures ANOVA (session by limb) was used to examine changes in CME as an effect of the training session on the two limbs (paretic and nonparetic). Significant main effects and interactions were followed up with *t*-tests corrected for multiple comparisons. Participants were classified as “responders” based on change in the paretic TA CME after training. A participant who showed a change of +5% compared to baseline was considered as a responder and the number of responders for each session are reported. A correlation analysis between change in CME of the P TA during the e-tDCS-HIITT and HIITT sessions was conducted to determine whether a relationship between the extent of response to HIITT alone was related to the extent of change during e-tDCS-HIITT. Statistical significance was set at *p* < 0.05. Greenhouse-Geisser correction was used when data violated sphericity assumptions. Values are reported as mean ± SE (standard error).

## 3. Results

**Figure 2:**
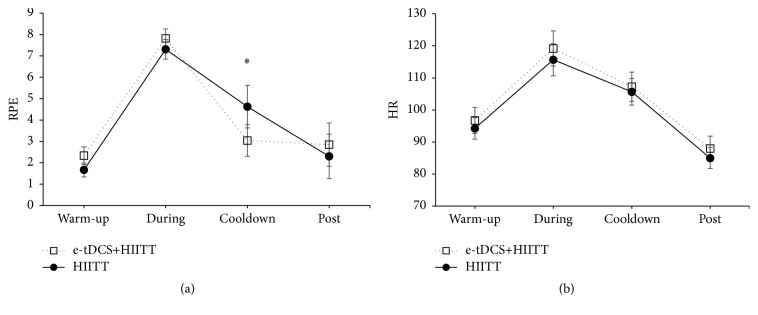
Rating of perceived exertion (RPE) and heart rate (HR; beats per minute (bpm)) are shown in Figures 2(a) and 2(b), respectively. *y*-axis represents 4 different time points at which these measurements were taken. Data are means and error bars are standard errors. There was a significant difference between the two sessions during cooldown for the RPE. A significant effect of time was noted for HR. ^*∗*^ *p* < 0.05.

All participants completed the training. No adverse effects of e-tDCS or treadmill training were reported. On average, participants started at a treadmill belt speed of 2.0 ± 0.12 mph and most were able to increase and/or maintain their speeds for 5–7 intervals. The highest belt speed achieved was on average 2.6 ± 0.16 mph. Details of treadmill speeds, number of intervals, and distance covered by each participant are provided in Table 2. Two-way repeated-measures ANOVA on RPE revealed a significant interaction for RPE (*F*_3,24_ = 4.24, *p* = 0.015). Paired *t*-tests for RPE at each level of time to compare the two sessions revealed a significant difference (*p* = 0.05) during “cooldown” (Figure 2(b)). On average, RPE increased from 2 during warm-up to 8 during the speed intervals. The RPE remained slightly elevated at 4.62 for the HIITT session compared to 3.04 for the e-tDCS+HIITT session. Two-way repeated-measures ANOVA on HR revealed a significant main effect of time for HR. HR changed similarly across both sessions and was found to be significantly different between the four time points (*F*_3,27_ = 101.768, *p* < 0.0001, Figure 2(a)). All participants were able to complete their treadmill training sessions without a need to discontinue.

### 3.1. Overground Gait Speed

No significant main effects or interactions were noted for overground gait speeds. Baseline gait speeds were similar for both sessions and a trend for improvement (change of 0.04 m/s) was noted after treadmill training (Table 3).

### 3.2. Change in Corticomotor Excitability

No significant differences were noted for the baseline MEP slopes for the NP TA (*p* = 0.38) and P TA (*p* = 0.43) between the two sessions. Differences were found between the baseline slopes for the NP and P TA within each session. The mean baseline NP TA slope was significantly steeper than the mean P TA slope for the HIITT (73%, *p* = 0.016) and e-tDCS+HIITT (79%, *p* = 0.013) sessions. Excellent ICC values were found between the baseline values of the NP TA (*R* = 0.83, *p* < 0.05) and the P TA (*R* = 0.94, *p* < 0.05) between both sessions. The average interhemispheric symmetry ratio for both baseline sessions was 0.49 ± 0.36, establishing a tendency towards an imbalance in interhemispheric symmetry for all participants.

**Figure 3:**
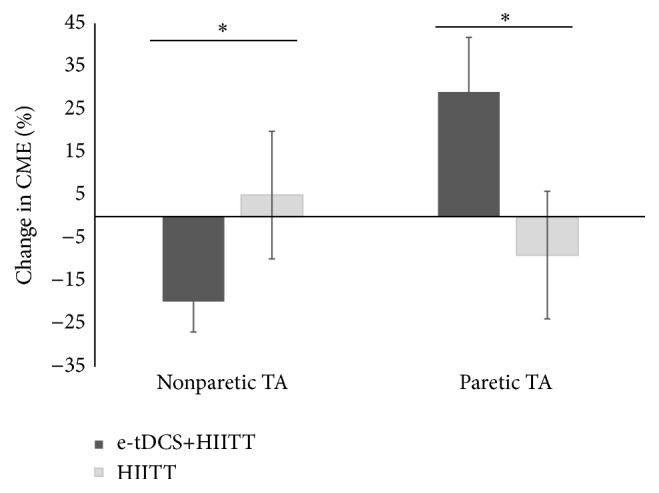
Percentage change in corticomotor excitability (CME) for the e-tDCS+HIITT (dark bars) and HIITT (light bars) groups. Corticomotor excitability was examined by calculating the change in linear slopes of the TMS recruitment curve before and after training for the bilateral tibialis anterior (TA) muscles. There were significant differences between the groups for the nonparetic and paretic TA muscles. ^*∗*^ *p* < 0.05.

**Figure 4:**
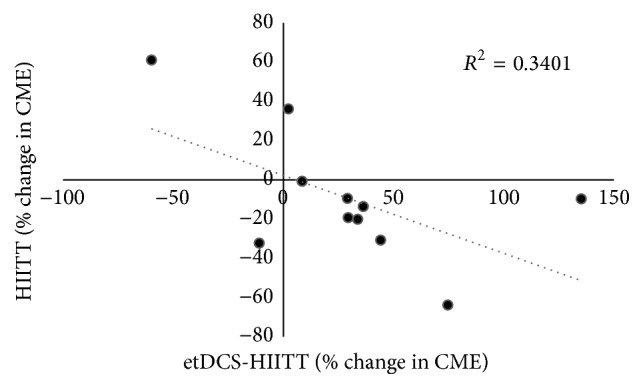
Relationship between percentage changes in corticomotor excitability of the paretic tibialis anterior muscle between the two sessions for all participants. Dashed line represents the negative linear relationship (*R* ^2^ = 0.34, *p* = 0.05) suggesting that those who were facilitated during e-tDCS+HIITT showed the most inhibition during HIITT alone.

The two-way ANOVA revealed a significant session × limb interaction (*F*_1,10_ = 5.647, *p* = 0.039). Post hoc analyses with corrected paired *t*-tests to compare the two sessions within each limb revealed significant effects for both the NP TA (*p* = 0.04) and P TA (*p* = 0.05) (Figure 3). For the NP TA, a decrease in CME was revealed following the e-tDCS+HIITT session (−19 ± 7%) compared to the increase in CME for the HIITT session (5 ± 11%). There was an increase in CME for the P TA following the e-tDCS+HIITT session (29 ± 14%) compared to a decrease in the HIITT session (−9 ± 37%). Single sample *t*-tests were used to check whether CME means differed from zero. The change in CME for the NP TA and P TA in the e-tDCS+HIITT group differed significantly from zero (*p* = 0.027 and 0.023, resp.). The change was not significant in either limb following HIITT alone. For the latter, small changes in means were accompanied by large variances (NP TA 5 ± 11%; P TA −9 ± 37%). Inspection of data revealed that only 2 of 11 participants had increased their P TA CME after HIITT, while the remaining 9 had decreased P TA CME. The mean reduction for the P TA for these 9 participants was −22 ± 6%, which differed from zero (*p* = 0.004). In the e-tDCS+HIITT group, 8 out 11 participants were classified as responders. The mean increase for the P TA for these 8 participants was 48 ± 13%, which differed from zero (*p* < 0.005). With all participants' data included, there was a negative correlation between reduced P TA CME during HIITT alone and change in CME during e-tDCS+HIITT (*R* ^2^ = 0.34, *p* = 0.05) (Figure 4).

## 4. Discussion

The objective of this study was to investigate the short-term effects of a single session of HIITT on corticomotor excitability in individuals with chronic stroke, and whether this modulation is affected by priming the motor cortex with tDCS and an ankle-tracking task prior to the exercise. HIITT alone resulted in small mean decreases in CME of the paretic TA and small mean increases in CME of the nonparetic TA, but the changes were not statistically significant. However, HIITT alone decreased CME in the majority of participants (9 of 11). When HIITT was preceded by the priming protocol (e-tDCS+HIITT), significant modulation was observed. After training, e-tDCS+HIITT induced an increase in CME of the paretic TA and a corresponding decrease in CME of the nonparetic TA. Interestingly, the correlation analysis revealed that participants with a greater CME downregulation also upregulated CME to a greater extent following priming with e-tDCS, indicating a possible shared neuroplastic mechanism. Both HIITT and e-tDCS+HIITT sessions showed a trend towards improved overground gait speeds (change of 0.03 and 0.09 m/s, resp.) after training. However, the change in gait speeds did not differ statistically between the two sessions.

This is the first study to examine the effects of HIITT on corticomotor excitability after stroke. Pohl et al. [3] and Sullivan et al. [4] independently investigated the effects of training at high treadmill velocities and demonstrated significant improvements in overground walking speeds with speed-dependent treadmill training compared to conventional therapy or at slow speeds. Faster speeds have also been shown to facilitate a more normal walking pattern after stroke without concomitant increases in common gait compensations, such as circumduction [5, 21]. Walking at progressively higher speeds not only requires increased cardiovascular activity but also increases neuromuscular demands to maintain the continuous stepping.

Another finding of this study is that CME of the paretic lower limb was augmented following e-tDCS+HIITT compared to HIITT alone. The fact that these changes were observed with a single session of training is promising and adds to the literature that forms the basis for investigating the effects of long-term training using cortical priming. An increase in CME of the P TA muscle was revealed following cortical stimulation-enhanced treadmill training. Interestingly, this increase in paretic TA CME was most evident in those who responded to HIITT with a downregulation of their paretic TA CME. The finding could indicate that exercise-induced neuroplastic mechanisms in some individuals make them candidates for motor priming protocols such as tDCS. Whether this acute increased neural drive is a predictor for long-term functional improvement is an interesting and important question for future studies.

The upregulation of CME in the e-tDCS+HIITT group was also accompanied by a decrease in CME of the NP TA. These results are consistent with previous studies that have revealed downregulation of the nonlesioned hemisphere along with an upregulation of the lesioned hemisphere [14, 16, 22]. There is support for the idea that upregulation of CME in the lesioned hemisphere is associated with a concomitant downregulation of the nonlesioned hemisphere via interhemispheric inhibition [9, 23]. Therefore, many upper limb studies have used noninvasive brain stimulation to suppress the nonlesioned hemisphere to produce an opposite modulation in the lesioned hemisphere (see [24] for review). However, in this pilot study, we did not provide behavioral data to support that the increase in paretic TA and decrease in the nonparetic TA are a positive functional outcome. Our hypothesis is supported by previous studies which have shown that a balanced CME is associated with less impairment and better function in stroke survivors [25–27].

The increase in neural excitability was not associated with a concomitant increase in gait speed. This is not surprising as it is unlikely that a single session of treadmill training would produce significant improvements in overground walking. Nevertheless, the trend towards improvement in gait speed is concurrent with other long-term training studies that have reported improvements in gait speeds with treadmill training [26, 28, 29].

RPE during cooldown from e-tDCS+HIITT was observed to be significantly lower than RPE from HIITT alone. RPE data were collected to detect the effects of tDCS on perceived exertion. The time course of RPE changes from warm-up to cooldown were similar for HIITT and e-tDCS+HIITT sessions. The slightly lower cooldown RPE mean for e-tDCS+HIITT may indicate a more rapid decrease in RPE for the e-tDCS+HIITT than the HIITT session. Further elegant physiological studies are needed to corroborate this finding because the primary outcome measure of this study is related to CME.

### 4.1. Limitations

Because this was a preliminary study, it has several limitations. First, a homogenous sample that we had previously found to have MEPs in the paretic TA was recruited. In addition, the people in this sample of stroke survivors were relatively fast ambulators (average gait speed of 0.93 m/s) who had the necessary strength and endurance to complete the HIIT protocol. These are unavoidable limitations. Second, the sample size was small (*n* = 11) and only a subset of this sample provided support for our hypothesis. Another study with a larger sample size would be necessary to confirm our results, and the inclusion of a measure of oxygen consumption may answer the question not addressed in the present study of whether the suppression of CME we observed in some of our participants was the result of greater physiological effort. Third, to avoid making the study unwieldy, we chose to compare the effects of HIITT alone with our robust tDCS+tracking task. The absence of tDCS-only and tracking-only control conditions prevents us from understanding which element of the intervention promoted the reduction in asymmetry of between-hemisphere CME. Hence, our results should be interpreted with caution. Fourth, we did not compare our results with other excitability boosting priming paradigms or to a standard form of therapy. Regardless of these limitations, our results are intriguing and can be used to support future studies that explore the impact on stroke survivors' motor function that might result from a HIITT-induced suppression of corticomotor excitability.

## 5. Conclusions

This is the first study to report that a single session of HIITT has the potential to exacerbate suppressed corticomotor excitability of paretic lower limb muscle representations in some individuals with stroke. Future studies are needed in order to optimize gait rehabilitation by examining the effectiveness of repetitive long-term HIITT with and without the inclusion of cortical excitability enhancing protocols.

## Figures and Tables

**Table 1:** Baseline characteristics of participants.

	Mean (SEM)
Age (years)	58 (2.7)

Height (cm)	165 (2.7)

Weight (lbs)	178 (9.4)

Time after stroke (years)	9 (1.8)

Type of stroke	
Ischemic	7
Hemorrhagic	4

Gender	
Male	4
Female	7

Affected limb	
Right	7
Left	4

Fugl-Meyer	
Paretic	23.6 (0.6)
Nonparetic	29.4 (0.3)

MMSE	28 (0.7)

**Table 2:** Treadmill speeds and distance covered for each participant during treadmill training.

	Overground gait speed (m/s)	e-tDCS+HIITT				HIITT			
		First interval (mph)	Last interval (mph)	Number of intervals	Total distance (miles)	First interval (mph)	Last interval (mph)	Number of intervals	Total distance (miles)
1	1.08	2	2.4	7	2.95	2.3	2.7	6	2.24
2	0.47	1.8	2.6	7	2.1	1.7	2.3	6	1.99
3	0.42	1.1	1.4	5	1	1	1.4	7	0.78
4	0.84	2	2.4	5	1.22	2	2.4	4	1.26
5	0.69	2	2.6	6	2.29	2	2.6	5	1.53
6	0.86	1.7	1.7	5	1.7	1.7	2.1	4	1.24
7	0.72	1.9	2.3	5	1.63	1.9	2.3	5	1.21
8	1.29	2.3	2.5	5	2.56	2.3	2.5	5	2.63
9	1.08	2.4	2.8	6	2.69	2.4	2.8	5	2.31
10	1.15	2.3	2.5	5	1.11	2.3	2.9	5	2.20
11	1.37	2.4	2.8	5	2.68	2.4	2.8	5	2.74

m/s: meters/second; mph: miles per hour.

**Table 3:** Overground fast walking speed.

Mean (SEM) gait velocity in m/s	Pre	Post

e-tDCS+HIITT	0.93 (0.09)	1.02 (0.09)
HIITT	0.93 (0.08)	0.96 (0.08)

m/s: meters/second.

## Additional Points

*Highlights.* (i) Corticomotor excitability (CME) following high intensity interval treadmill training (HIITT) has not been examined in stroke. (ii) A single session of HIITT reduces CME of the lesioned hemisphere in some stroke survivors. (iii) Motor priming with task-enhanced transcranial direct current stimulation (tDCS) prior to HIITT reduces this suppression and improves the between-hemisphere symmetry of CME.
